# Cyclooxygenase 2-dependent and independent activation of Akt through casein kinase 2α contributes to human bladder cancer cell survival

**DOI:** 10.1186/1471-2490-11-8

**Published:** 2011-05-18

**Authors:** Keiji Shimada, Satoshi Anai, Develasco A Marco, Kiyohide Fujimoto, Noboru Konishi

**Affiliations:** 1 Department of Pathology, Nara Medical University School of Medicine, Shijo-cho 840, Kashihara city, Nara, 634-8521; Japan; 2 Department of Urology, Nara Medical University School of Medicine, Shijo-cho 840, Kashihara city, Nara, 634-8521; Japan; 3 Department of Urology, Kinki University School of Medicine, Sayama city, Osaka, 589-8511, Japan

**Keywords:** cyclooxygenase 2, urothelial carcinoma, casein kinase 2α, Akt

## Abstract

**Background:**

Survival rate for patients presenting muscle invasive bladder cancer is very low, and useful therapeutic target has not been identified yet. In the present study, new COX2 downstream signals involved in urothelial carcinoma cell survival were investigated *in vitro *and *in vivo*.

**Methods:**

COX2 gene was silenced by siRNA transfection. Orthotopic implantation animal model and transurethral instillation of siRNA with atelocollagen was constructed to examine the effects of COX2 knockdown *in vivo*. Cell cycle was examined by flowcytoketry. Surgical specimens derived from patients with urinary bladder cancer (all were initially diagnosed cases) were used for immunohistochemical analysis of the indicated protein expression in urothelial carcinoma cells.

**Results:**

Treatment with the COX2 inhibitor or knockdown of COX2 reduced expression of casein kinase (CK) 2 α, a phophorylated Akt and urokinase type plasminogen activator (uPA), resulting in p27 induction, cell cycle arrest at G1 phase and cell growth suppression in human urothelial carcinoma cell lines expressing COX2. Silencing of CK2α exhibited the similar effects. Even in UMUC3 cells lacking the COX2 gene, COX2 inhibition also inhibited cell growth through down-regulation of the CK2α-Akt/uPA axis. The mouse orthotropic bladder cancer model demonstrated that the COX2 inhibitor, meloxicam significantly reduced CK2α, phosphorylated Akt and uPA expression, whereas induced p27 by which growth and invasiveness of bladder cancer cells were strongly inhibited. Immunohistochemically, high expression of COX2, CK2α and phosphorylated form of Akt was found in high-grade, invasive carcinomas as well as carcinoma *in situ*, but not in low-grade and noninvasive phenotypes.

**Conclusions:**

COX2-dependent and independent activation of CK2α-Akt/uPA signal is mainly involved in urothelial carcinoma cell survival, moreover, not only COX2 but also CK2α could be direct targets of COX2 inhibitors.

## Background

Cyclooxygenase (COX) 2 is an inducible enzyme that produces prostaglandins during inflammatory and tumorigenic processes[1]. The biology of COX2 in relation to tumor genesis has been well studied, particularly with regard to colorectal cancer development. Aberrant expression of COX2 as well as deregulation of WNT/β-catenin signaling occurs in the majority of colorectal tumor[2,3]. Deregulation of COX-2 expression leads to an increased abundance of eicosanoids that affect the hallmarks of cancer. For examples, COX2/prostaglandin E2 signal is thought to protect tumor cells or tumor initiating cells from apoptosis induction by regulating pro- and/or anti-apoptotic molecules[4,5]. In contrast, studies using *in vivo *animal models clearly indicated over expression or over activation of COX2 only cannot develop tumors spontaneously: breast cancer or colon cancer cannot be induced in COX2 transgenic mice without a murine mammary tumor virus infection or azoxymethane treatment[6,7]. Thus, COX2 may be associated with promotion, but not initiation, of several types of human cancer.

Urinary bladder cancer is a common malignancy in industrialized countries including Japan. More than 90% of bladder cancer originates in the urothelial (transitional) cells[8]. Low grade urothelial carcinoma can be usually controlled by intravesical therapy; in contrast, high grade cancer is much difficult to treat. Therefore, identification of target molecules or signals involved in urothelial carcinoma with high malignant potential is needed for successful therapy. COX2 is well known to enhance malignant potential of urothelial carcinoma cells[9,10], and several types of COX2 inhibitors are clinically used for not only treatment but also prevention of bladder cancer[11]. However, COX2-mediated signals involved in urothelial carcinoma cell survival remain fully undetermined, in addition, the fact that COX2 inhibitors have both COX2 -dependent and -independent cytotoxic effects make us much difficult to understand the biological roles. Casein kinase (CK) 2 is a ubiquitous serine/threonine protein kinase, and its heterotetrameric structure consists of two catalytic subunits (~42 kDa αand 38 kDa α') and two regulatory subunits (~28 kDa β) in α2β2, αα'β2, or α'2β2 configurations[12]. Recent reports demonstrated CK2 is closely associated with tumor progression by phosphorylating a number of kinases [13,14]. CK2 is widely expressed in various types of malignant tumors[14,15], and many investigators have focused on CK2 as a therapeutic target. Wang H. et al. showed CK2 down-regulation induced apoptosis in prostate cancer cells that may lead to novel cancer therapies[16]. However, there are no available data on the clinicopathological significance of CK2α in bladder cancer. In the present study, we found that COX2 is an upstream molecule of CK2α leading to Akt activation and urokinase type of plasminogen activator (uPA) induction. Silencing or inhibition of COX2 successfully inhibits CK2α-Akt/uPA axis, resulting in cell cycle arrest and growth suppression of bladder cancer cells *in vitro *and *in vivo*. Interestingly, even in urothelial carcinoma cells lacking COX2 gene, COX2 inhibitors reduced expression of CK2α and cell viability. The data suggest both COX2 dependent and independent activation of CK2α-Akt/uPA-mediated promotion and such signals could be targeted by COX2 inhibitors in human bladder cancer.

## Methods

### Bladder cancer cell lines, chemicals and antibodies

Human urothelial carcinoma cell lines UMUC2 and UMUC3 were cultured in RPMI supplemented with 10% fetal bovine serum. The origins of UMUC2 and UMUC3 are urethral tumor (carcinoma *in situ*) and bladder tumor (invaded carcinoma), respectively. Wild type of p53 is observed in UMUC2 cells, but it is largely deleted in UMUC3 cells[17]. KU7 was derived from human papillary bladder cancer[18]. We generated stable green fluorescent protein (GFP) KU-7 cells as previously described[19,20]. Antibodies to COX2, p27, Akt and phosphorylated form Akt were purchased from Cell Signaling (Boston, MA, USA); Antibodies to actin and COX1 were from Santa Cruz Biotechnology (Santa Cruz, CA, USA); Antibodies to CK2α were supplied by Abcam (Cambridge, UK). COX2 selective inhibitor, CAY10404 or DuP-697 was from Cayman (Ann Arbor, USA). Phosphatidylinositol-3-kinase inhibitor, LY294002, soluble in DMSO was purchased from Cell Signaling Technology Japan (Tokyo, Japan).

### Preparation of cell lysates and Western blotting analysis

Cell lysates were resolved in SDS-polyacrylamide gels and transferred onto polyvinylidene difluoride membranes (Millipore, Ltd., MA, USA), which were then blocked in 5% skim milk at room temperature for 1 h. Membranes were incubated with the indicated primary antibody (1:50-1000 dilution according to the manufacture's protocol) for 1 h, and then incubated with horseradish peroxidase-conjugated antimouse or antirabbit IgG (1:5000 dilution) (Amersham Pharmacia Biotech, Boul Morgan Baie-D'Urfe, Canada). We detected peroxidase activity on X-ray films using an enhanced chemiluminescence detection system.

### Reverse transcription-PCR

Using the One Step RT-PCR kit (Qiagen), we extracted total RNA using Trizol reagent and subjected it to reverse transcription-PCR (RT-PCR). PCR conditions were 95°C for 30 s, 55 to 58-60°C for 30 s, and 72°C for 1 min through a total of 30 cycles.

The PCR primer sequences for uPA were 5'-TCACCACCAAAATGCTGTGT-3' (sense) and 5'-AGGCCATTCTCTTCCTTGGT-3'(antisense). The primers for COX2 were 5'-GCAATAACGTGAAGGGCTGT-3' (sense) and 5'-CGGGAAGAACTTGCATTGAT-3' (antisense). For glyceraldehyde-3-phosphate dehydrogenase (GAPDH), the primers used were 5'-ACCACAGTCCATGCCATCAC-3' (sense) and 5'-TCCACCACCCTGTTGCTGTA-3' (antisense).

### siRNA transfection of COX2/CK2α

For our transfection analyses, 10^6 ^cells from each bladder cancer cell line were seeded in 6-cm dish plates and transfected either with 100 nmol/L of control siRNA (Santa Cruz Biotech., CA, USA) or with the siRNA of COX2, CK2α. Transfections were carried out using the Lipofectamine system (Invitrogen, CA, USA) in accordance with the manufacturer's protocol. COX2, CK2α siRNA duplexes, generated with 3'-dTdT overhangs and prepared by Qiagen, were chosen against the DNA target sequences as follows: 5'-AACACCGGATTTTTGACAAG-3' for COX2; 5'-CTGGTCGCTTACATCACTTTA-3' and 5'-TCCATTGAAGCTGAAATGGTA-3'for CK2α (cocktail).

### Analysis of uPA activity

uPA activity in culture media was analyzed using an uPA activity assay kit (Chemicon International, Billerica, MA, USA). In accordance to the manufacture's protocol, culture media were collected after indicated treatments or siRNA transfection. Media were concentrated by centrifugation, samples were added into 96-well plate together with positive control, and reacted with several indicated reagents including chromogenic substrates. After incubation at 37°C for 30 min, absorbance was measured on a standard microplate reader at 405 nm. All experiments were done at least thrice in triplicate.

### Cell cycle analysis

We performed cell cycle analysis using propidium iodide by flow cytometry as previously described [22]. All experiments were done at least thrice in duplicate.

### Orthotropic tumor implantation and intravesical treatment

Animal experiments were approved by the institutional animal care and use committee at Nara Medical University. Eight week-old female nude mice were maintained on a daily 12-h cycle of light and dark and were fed standard diet and water *ad libidum*. Orthotropic bladder cancer exhibiting muscle invasive phenotype was established by inoculating KU7/GFP cells (5 × 10^6^) into the mouse bladder using transurethral catheter[19]. Seven days after tumor cell inoculations, mice were randomized into control (n = 6) or meloxicam (n = 6), treatment groups. Before the beginning of treatment with meloxicam, we captured *in vivo *images of tumor masses in the urinary bladder, and identified tumor implantation there were no significant differences of fluorescence intensities between control and drug treatment groups. Meloxicam was fed in the drinking water (3.0 mg/l). Mice were sacrificed 25 d after treatment and bladders were removed, splayed open on filter paper and fixed in 10% neutral buffered formalin. Tumor burden was determined by analyzing images of GFP fluorescence from tumor cells on flat formalin-fixed bladders captured using a macro-imaging station consisting of a SBIG cooled CCD camera model ST-7XME (Santa Barbara Imaging Group Inc., Santa Barbara, CA, USA) mounted onto a dark box. Bladders were embedded in paraffin, step-sectioned, stained with hematoxylin and eosin (H&E) for histological evaluation. Invasion depth means the length from the top to the bottom level of the largest tumor. Results from animal experiments were compiled from data generated from three independent experiments. RNA and protein extractions were performed as follows: Tumor masses expressing GFP was removed under the stereoscopic fluorescence microscopy and the tumors were fragmented by shaver. RNA was extracted by TriReagent (Ambion, Austin, Texas, USA) according to the manufacturers' instructions. Protein was extracted after homogenization using lysis buffer.

### Image analysis

A method for the quantification of tumor burden by computer assisted image analysis has been described elsewhere [19]. Briefly, image analysis was performed with ImageJ public domain software available through the National Institutes of Health (Bethesda, MD; available at http://rsb.info.nih.gov/ij/). All images were spatially calibrated for area measurements. Signal strength was recorded as electrons emitted per second of exposure. The area under the curve (AUC) was determined from plot profiles based on fluorescent signal strength and distribution in each individual bladder.

### Cell viability assay (MTS assay)

After incubation with the indicated reagents, MTS [(3-(4,5-dimethylthiazol-2-yl)-5-(3-carboxymethoxyphenyl)-2-(4-sulphonyl)-2H-tetrazolium, inner salt] reagent (Promega, Tokyo, Japan) was added and optical absorbance at 490 nm was measured using a microplate reader. Cell viability was expressed as mean percentages the standard deviations of absorbance before and after treatment with various reagents. All experiments were performed in triplicate.

### Tissue samples and immunohistochemistry

We obtained specimens of human urinary bladder cancers diagnosed as urothelial carcinomas (*n *= 93) from patients undergoing transurethral resection or radical cystectomy, without previous radiation or chemotherapy, at Nara Medical University Hospital. Clinicopathologic data of the present cases were reviewed by two urological pathologists (K.S. and N.K., department of Pathology, Nara Medical University Hospital) and summarized in Table 1. The current research was approved by institutional research board of Nara Medical University and informed consent was obtained from all patients. Before the collection of specimens, as appropriate, and tumor stage and grade were noted at the time of diagnosis. We followed the same tissue fixation and processing procedure as described in a previous report[21,22]. After deparaffinization, sections were heated for 5 minutes in 10 mM of sodium citrate buffer (pH 6.0) in a pressure cooker. The sections were then incubated overnight at 4°C with the indicated antibodies. The reactions were visualized using a Histofine SAB-PO kit and diaminobenzidine as the chromogen (Nichirei, Tokyo, Japan) with hematoxylin counterstaining. Percentages of cells positive for CK2α, COX2 or phosphorylated form of Akt were expressed per 1,000 cells examined.

**Table 1:** Characterization of urothelial carcinomas

Mean Age (years)	72.2(44 to 96)
Gender	70:23(Male:Female)

Pathological stage	
pTis	14(15%)
pTa	29(31%)
pT1	27(29%)
≧pT2	23(25%)

Grade	
Low grade	51(55%)
High grade	42(45%)

### Statistical analysis

Data were statistically analyzed using the Student *t *test or, for nonparametric analysis, the Kruskal-Wallis test[23]. Results were considered significant if *P *< 0.05.

## Results

### Inhibition of COX2 suppressed growth of urothelial carcinoma cells

**Figure 1:**
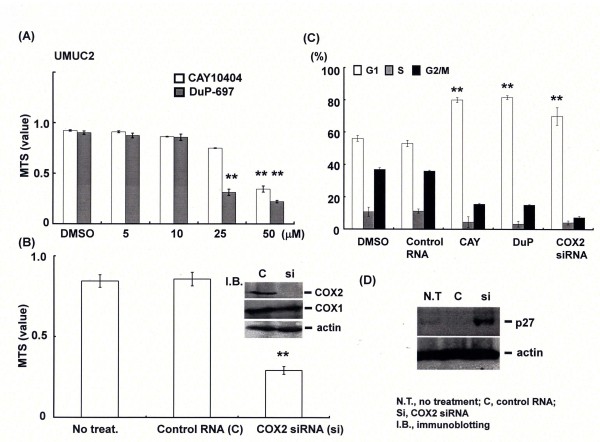
**COX2 inhibition or the gene silencing induced cell cycle arrest and cell growth suppression**. (A) UMUC2 cells were treated by the selective COX2 inhibitor, CAY10404(CAY) or DuP-697(DuP) at the indicated concentrations for 48 h, then cell viability was assessed by MTS assay. (B) At 72 h after transfection of control or COX2 siRNA at 100 nM, cell viability and COX2/COX1 expression were analyzed by MTS assay and western blotting, respectively. Actin was used for internal positive control. (C) and (D) UMUC2 cells were transfected by control or COX2 siRNA, or treated by DMSO, CAY or DUP, then cell cycle analysis was performed by flow cytometry and p27 expression was examined by western blotting. Dimethyl sulfoxide was used as control reagent.

COX2 inhibitors, DuP-697 and CAY10404 at more than 25 μM significantly suppressed viability of human urothelial carcinoma cell line, UMUC2 expressing wild type COX2 as assessed by MTS assay (Figure 1(A)). COX2 activities in cancer cell lines were strongly inhibited by these treatments (data not shown). COX2 gene silencing by siRNA transfection exhibited the same effects (Figure 1(B)). Flow cytometry analysis showed both COX2 inhibitors treatment and COX2 knockdown induced cell cycle arrest at G1 phase. From western blotting, p27 was found to be induced in response to COX2 gene silencing (Figure 1(D)), but other molecules associated with cell cycle arrest including p53, p21, p16 or Rb protein were not significantly modified (data not shown).

### COX2-dependent activation of casein kinase/uPA signal in urothelial carcinoma cells

**Figure 2:**
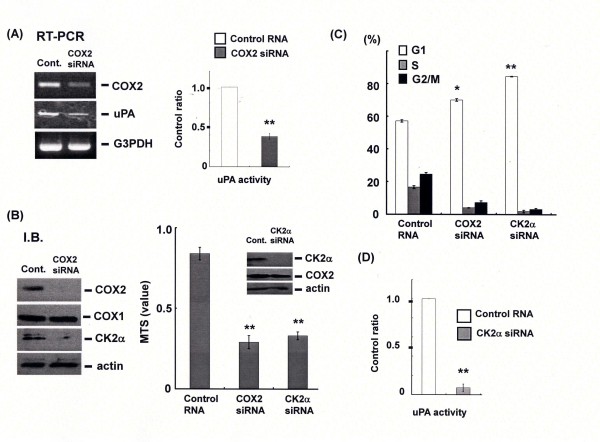
**CK2α and uPA are downstream of COX2 in UMUC2 cells**. (A), (B) and (D) UMUC2 cells were transfected by control or siRNA of COX2 or CK2α. After 72 h-incubation, mRNA and protein of COX2/COX1, uPA or CK2α were examined by RT-PCR and western blotting. Activity of uPA in culture media was assessed. Cell viability was analyzed by MTS assay. (C) Cell cycle analysis was performed by flow cytometry. Dimethyl sulfoxide was used as control reagent.

Recently, plasminogen activator systems have been shown to play important roles in the development of urinary tumors[24]. Therefore, we examined whether urokinase or tissue type of the plasminogen activator was regulated by COX2 in urothelial carcinoma cells. Figure 2A clearly demonstrated that mRNA expression and activity of uPA were down-regulated in response to COX2 knockdown (Figure 2A). In addition, we found that COX2 contributes to the expression of CK2α, an upstream molecule of uPA and G1 cell cycle arrest was induced by silencing the CK2α gene as well as COX2 (Figures 2B,C and 2D).

### COX2 affects CK2α-mediated activation of Akt in urothelial carcinoma cells

**Figure 3:**
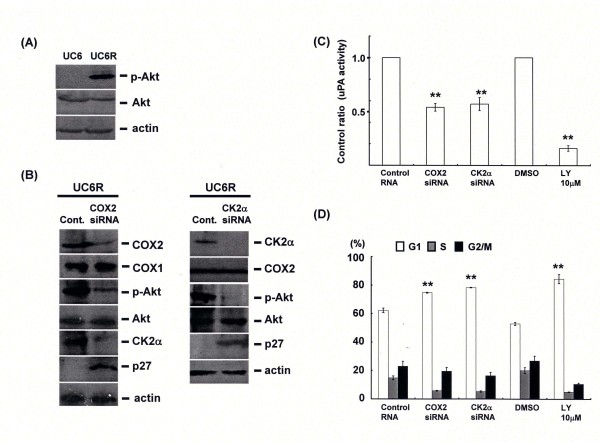
**COX2 regulates Akt activation through CK2α in UMUC6R cells**. (A) UMUC6R cells were obtained from parental line, UMUC6 by long-term incubation under media including doxorubicin. Phosphorylated Akt and Akt were examined by western blotting. (B), (C) and (D) UMUC6R cells were transfected by control siRNA or the indicated siRNA of COX2 or CK2α. After 72 h-incubation, expressions of the indicated gene were examined. Activity of uPA in culture media at 72 h after transfection of control siRNA or siRNA, or at 48 h after treatment with DMSO or specific inhibitor of Akt, LY294002 at 10 μM (for 30 min) was assessed. Cell cycle analysis was performed by flow cytometry. Dimethyl sulfoxide was used as control reagent.

Since activation of phosphoinositol 3 kinase-Akt signal is one of the upstream targets of plasminogen activator systems, we investigated association of COX2-CK2α pathway to Akt. In this experiment, we used a doxorubicin resistant clone derived from a human urothelial carcinoma cell line, UMUC6 that we have already constructed by long term culture using the medium including doxorubicin at the sub-lethal dose. Akt was found to be constitutively activated in the UMUC6 resistant (UMUC6R) clone (Figure 3A). Both COX2 and CK2α knockdowns inhibited Akt activation, but its baseline expression was not significantly changed (Figure 3B). Reduced uPA activity and cell cycle arrest at G1 phase through p27 induction was mediated by Akt inhibitor treatment as well as COX2 or CK2α gene silencing in UMUC6R cells (Figure 3C).

### COX2 inhibitor suppressed COX2- CK2α-Akt-uPA signal independent of COX2

**Figure 4:**
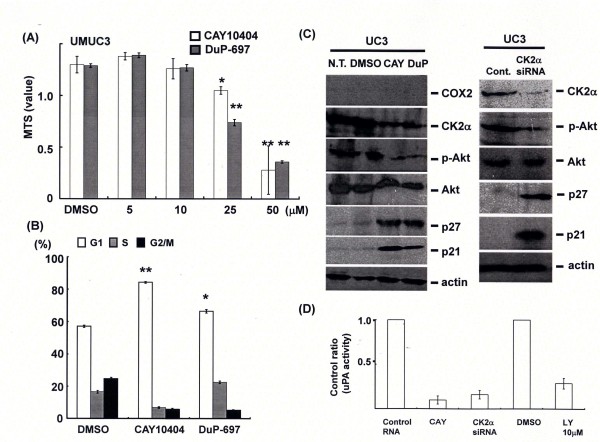
**Activation of CK2α-uPA signal independent of COX2 in urothelial carcinoma cells**. (A) UMUC3 cells lack COX2 gene were treated by selective COX2 inhibitor, CAY10404(CAY) or DuP-697(DuP) at the indicated concentrations for 48 h, then cell viability was assessed by MTS assay. (B) Cells were treated by DMSO, CAY or DuP at 25 μM for 48 h, and then Cell cycle analysis was performed by flow cytometry. (C) and (D) At 48 h after the same treatments (plus LY294002 treatment) or at 72 h after transfection of control or CK2α siRNA, protein expression (COX2, CK2α, phosphorylated Akt, Akt, p27 or p21) and activity of uPA were examined. Dimethyl sulfoxide was used as control reagent.

Tumor suppression by COX2 inhibitors without affecting COX2 expression or activity has attracted considerable attention lately. The human urothelial carcinoma cell line, UMUC3 lacks the COX2 gene, COX2 inhibitors, CAY10404 and DuP-697 significantly suppressed cancer cell growth in a dose dependent manner through the induction of cell cycle arrest at G1 phase (Figure 3A and 3B). Expression of CK2α and phosphorylated Akt was down-regulated by COX2 inhibitors and knockdown of CK2 (Figure 4C). Interestingly, uPA activity was reduced by COX2 inhibitors, and p21 was induced in response to COX2 inhibitors treatment or CK2α gene silencing in addition to p27.

### *In vivo *growth of urothelial carcinoma is suppressed by COX2 inhibitor, Meloxicam through affecting CK2α-Akt-p27 signals

**Figure 5:**
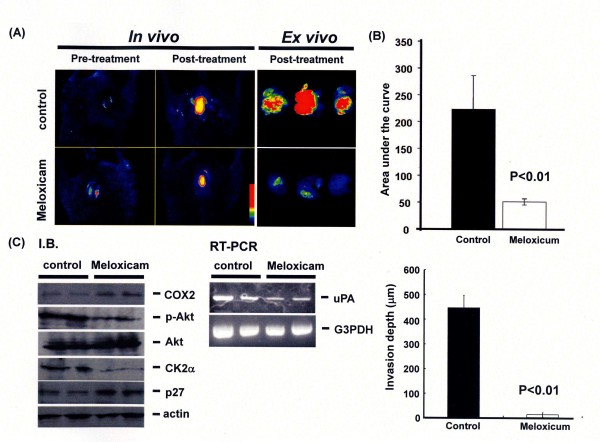
**COX2 inhibitor suppressed tumor growth through down-regulating CK2α-Akt and uPA signals *in vivo***. (A) and (B) KU-7-GFP (constitutively over expression of GFP) cells were transplanted into the urinary bladder of nude mice. At 7 days after inoculation, mice were randomized into a control (n = 6), meloxicam (n = 6), treatment groups. COX2 inhibitor, meloxicam was fed (3.0 mg/l). Mice were sacrificed 25 days after treatment; tumor size was quantitatively analyzed by using *in vivo *and *ex vivo *imaging. (C) mRNA or protein were extracted from the tumor samples and expression of COX2, phosphorylated Akt, Akt, CK2α, p27 or uPA was examined by western blotting or RT-PCR (left panel). Invasion depth from the top to bottom of implanted tumor foci was measured (right panel).

KU-7 cells stably expressing the GFP encoding vector were inoculated into the urinary bladder using a transurethral catheter (Day0). Seven days after inoculation, we determined grafting to the bladder wall by a fluorescence imaging technique and mice were randomized into a control (n = 6) or meloxicam (n = 6), treatment groups. Meloxicam fed in the drinking water (3.0 mg/l) and mice were sacrificed 25days after treatment. As shown in Figures 5A and 5B, *in vivo *and *ex vivo *images showed meloxicam treatment produced a 5-fold decrease in tumor area. Invasion depth from the top to bottom of implanted tumor foci was strongly reduced by COX2 inhibitor treatment. Consistent with these data, in the treatment group, mRNA of uPA and protein levels of CK2α or phosphorylated form of Akt were significantly reduced, in contrast, p27 was up-regulated. In KU-7-GFP cells, seldom expression of COX2 was observed and it was slightly up-regulated after meloxicam treatment *in vivo *probably due to a feedback effect.

### COX2, CK2α and activated Akt are over expressed in human urothelial carcinoma of the urinary bladder

**Figure 6:**
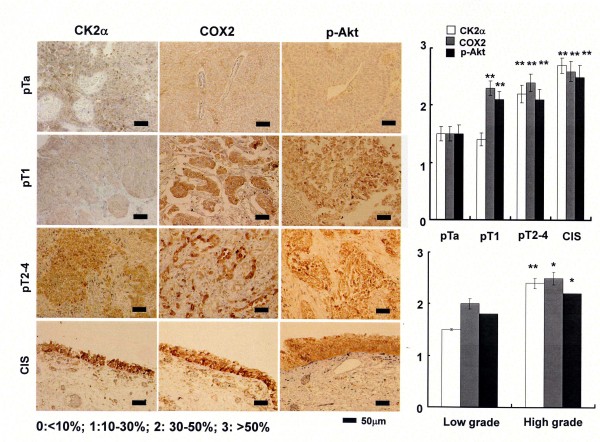
**Immunohistochemical analysis of COX2, CK2α and phosphorylated Akt in human bladder cancer**. Expression of COX2, CK2α or phosphorylated Akt was immunohistochemically analyzed. Positive percentages were calculated were classified into four intensities (0, less than 10%; 1, 10-30%; 2, 30-50%; 3, more than 50%). The mean of intensity levels in each stage (pTa, pT1, more than pT2 or CIS (pTis)), or in low or high grade tumor were indicated. Statistical differences of percentages of immunopositive cells between non-invasive/low grade cancer and invasive/high grade phenotype (* p < 0.05, **p < 0.01).

Finally, immunohistochemical analysis of COX2, CK2α or phosphorylated Akt was performed. We examined surgical specimens consisting of low-grade/noninvasive, high-grade/invasive, and carcinoma *in situ *(CIS), all of which were diagnosed by two urologic pathologists (K.S. and N.K.). As shown in Figure 6, the percentages of immunopositive cells for COX2 or activated Akt were much higher in high-grade urothelial carcinomas, including CIS, with both minimal (pT1) and wide invasion (≧pT2) than in low-grade (G1 and G2) lesions with a noninvasive phenotype (pTa). Immunohistochemical results of CK2α showed an almost similar tendency, but no significant difference of percentages was not observed between non-invasive (pTa) and minimal invasive (pT1) tumors (Figure 6 left upper panel). In normal urothelium obtained from autopsy sample, we found no or seldom expression of COX2, casein kinase 2α and activated Akt (data not shown). Taken together, over expression of COX2, CK2α and phosphorylated Akt is closely associated with bladder cancer progression but more *in vitro *experiments are required to know whether these markers are direct targets to turn less invasive cancer to a more invasive phenotype.

## Discussion

We demonstrated here for the first time that CK2α-meidated uPA signal through phosphorylated Akt is dependent on COX2 and plays an important role in cell survival of human urothelial carcinoma cells. To date, a number of reports have been accumulated as to the accelerating effects of COX2 on bladder cancer development, but to our knowledge, no investigations have pointed out the importance of CK2α as a downstream molecule. Manna et al. demonstrated the compound 5-(4-methoxyarylimino)-2-N-(3,4-dichlorophenyl)-3-oxo-1,2,4-thiadiazolidine (P(3)-25), which is known to possess anti-bacterial, anti-fungal, anti-tubercular, and local anesthetic activities, reduced phosphorylation of p65 by inhibiting upstream kinases including protein kinase A, CK2α etc resulting in down-regulation of nuclear factor (NF) kappaB-dependent reporter gene, COX2 in cancer cells with constitutive expression of NF kappaB[25]. Our current data indicated reduced expression of CK2α following COX2 inhibitor or the gene silencing, but phosphorylation of p65 was not observed (data not shown). Whether COX2 interacts with CK2α through NF kappaB-dependent or independent pathway should be estimated using malignant cells. The present study provided COX2/CK2α is an upstream of Akt phosphorylation in urothelial carcinoma cells. CK2 has been previously reported to increase association of Akt to the Heat shock protein (HSP) 90 which maintains stability of phosphorylated Akt at threonine 308 or serine 473 by inhibiting dephosphorylation by protein phosphatase2A[26,27]. Because expression of Akt was not affected by knockdown of COX2 or CK2α, CK2α-meditaed stability of Akt-HSP90 might be a main mechanism by which COX2 is closely related to Akt phosphorylation at threonine 308 in urothelial carcinomas. COX2 is fully expressed, but Akt is not constitutively activated, in UMUC2 cells, but it can be activated in response to specific external stimuli (data not shown), therefore, COX2/CK2α may not directly activate Akt but contributes to the maintenance of Akt once phosphorylated. COX2/CK2α and uPA signal actually functioned even in UMUC2 cells lacking activated Akt, therefore, CK2α is able to mediate uPA induction independent of Akt. The interesting data was that COX2 inhibitors down-regulated CK2α-Akt and uPA axis in urothelial carcinoma cells lacking the COX2 gene. Moreover, it is epoch-making that COX2 inhibitor can be used for bladder cancer therapy regardless of COX2 status at least in the cases with functional CK2α-Akt-uPA, even though it should be determined whether all COX2 inhibitors have the same pharmacological action. IC50 values of CAY10404 and DuP-649 for COX1 are 500 μM and 9 μM, respectively. Therefore, there are any possibilities that COX1 contributes to activation of casein kinase 2 alpha as well as COX2 in UMUC3 cells. Previous studies showed the importance of phosphatidyl inositol 3 kinase and Akt pathway in urothelial carcinoma[28,29], but there are no clinically available inhibitors of this pathway. Possibly we can substitute COX2 inhibitors in bladder cancer treatment. To make sure we examined the effects of COX2 inhibitors on cell survival in other cell lines used in the current study than UMUC2 and UMUC3. The results showed both CAY10404 and DuP-649 similarly suppressed cell survival of KU-7, UMUC6 and UMUC6R cells at the concentrations more than 25 μM (data not shown).

We found that the COX2 and CK2α-Akt-uPA signal actually function *in vivo *using the orthotropic implantation animal model. Oral feeding of COX2 inhibitor could dramatically reduced expression of CK2α, phosphorylated Akt and uPA, resulting in reduction of tumor growth and invasiveness. Unfortunately we have no data to explain the mechanism by which COX2 protein expression in KU7 cells implanted in the urinary bladder was increased in response to treatment with meloxicam. But this reaction is probably due to feedback regulation by inhibition of enzymatic activity of COX2, or due to execution of unidentified molecules or signals by COX2 inhibitors, resulting in transcriptional activation or protein stabilization. It should be further evaluated. Pathological analysis using human bladder cancer specimens clearly indicated that the COX2-CK2α and Akt phosphorylation axis is amplified in human urothelial carcinoma cells, particularly in advanced cases. These results mean that COX2 expression and Akt activation change urothelial carcinoma cells from non-invasive to an invasive phenotype. In addition to COX2/phosphorylated Akt, CK2α induction is essential for the progression to high grade and muscle invasive cancer. In urothelial carcinomas with microinvasion, CK2α signal does not function but Akt is activated, suggesting that Akt can be phosphorylated even in the absence of CK2α, but it is necessary for its sustainable activation and cancer development. We cannot explain why CK2α was not detectable in pT1 tumors, even though COX2 was expressed. Positivity and intensity of COX2 protein did not differ between tumors of pT1 and more than pT2, therefore, COX2 expression level is not essential. There is the possibility that the activity of COX2 or its products, including eicosanoids, might affect the transcription or stabilization of CK2α. Expression of COX2, CK2α or phosphorylated Akt in dysplasia was similar to that in high grade carcinoma in situ (n = 12, intensities of CK2α, COX2 and phosphorylated Akt were 2.2 ± 0.08, 2.0 ± 0.10 and 2.3 ± 0.07, respectively.). Urothelial dysplasia is preclinical cancer and low grade intraepithelial neoplasia, but its malignant potential is almost similar to high grade cancer from immunoprofiles of COX2, CK2α and phosphorylated Akt. CK2α and phosphorylated Akt signal might be useful targets for human bladder cancer treatment. The pathological significance of COX2 or Akt phophorylation has been well investigated independently[10,29,30], and the current study opened the window for the problem, 'how these factors interact for each other'. COX2 affects Akt activation through manipulating CK2α expression, which is mainly involved in bladder cancer development.

## Conclusions

**Figure 7:**
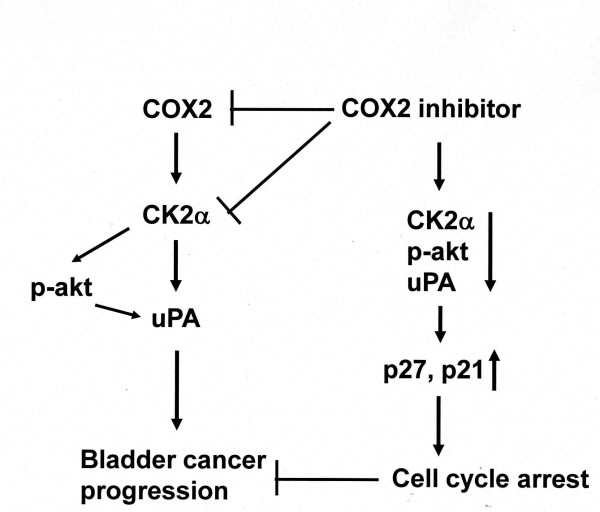
**Schematic presentation of COX2 dependent and -independent signals involved in bladder cancer development**. COX2-CK2α signal is associated with sustained activation of Akt. COX2 inhibitors suppress the COX2-CK2α-phosphorylated Akt and uPA signal, resulting in p27/p21 induction.

There are COX2-depdent and -independent mechanisms by which CK2α-phosphorylated Akt and uPA signal is activated, resulting in advancement of human bladder cancer. COX2 inhibitors can shut down not only COX2 but also CK2α level in this signal and bring more effective therapeutic outcome (Figure 7).

## Competing interests

The authors declare that they have no competing interests.

## Authors' contributions

KS and SA carried out *in vitro *experiments and immunohistochemical analysis using human tissue samples and performed the statistical analysis, moreover KS drafted the manuscript. DVM constructed animal model of orthtopic implantation of human bladder cancer cells, instillation of siRNA and image analysis of resected samples. KF and NK participated in the design of the study and coordination. All authors read and approved the final manuscript.

## Pre-publication history

The pre-publication history for this paper can be accessed here:

http://www.biomedcentral.com/1471-2490/11/8/prepub
